# Causal links and mediating effects of lipid metabolism, immune cells, and inflammatory proteins in endometriosis: Evidence from Mendelian randomization

**DOI:** 10.1097/MD.0000000000043163

**Published:** 2025-07-11

**Authors:** Pei Guo, Junhong Gan, Lin Xu, Weihong Li

**Affiliations:** a Graduate School, Guangxi University of Chinese Medicine, and Guangxi International Zhuang Medicine Hospital Affiliated to Guangxi University of Chinese Medicine, Nanning, Guangxi, China; b Faculty of Chinese Medicine Science, Guangxi University of Chinese Medicine, Nanning, Guangxi, China; c School of Pharmacy of Guangxi University of Chinese Medicine, Nanning, Guangxi, China.

**Keywords:** endometriosis, immune traits, inflammatory proteins, lipid metabolism, Mendelian randomization

## Abstract

Endometriosis (EMS) is a complex gynecological disorder whose pathogenesis remains poorly understood, with lipid metabolism, immune regulation, and inflammation likely playing pivotal roles. This Mendelian randomization study investigates causal relationships between lipid metabolic levels, immune cell characteristics, inflammatory proteins, and EMS using multi-omics data from 179 lipid metabolites, 731 immune cell traits, and 91 inflammatory proteins, combined with EMS cases from the FinnGen database. Sensitivity analyses were conducted using inverse variance weighted, Mendelian randomization Egger regression, weighted median, and weighted mode methods to ensure robust findings. Our analysis identified significant associations between 21 lipid metabolites and EMS, with 9 metabolites showing protective effects and 12 promoting risk. Specifically, triacylglycerol (46:2) levels displayed a reverse causal relationship with EMS. Additionally, 32 immune cell traits and 6 inflammatory proteins were linked to EMS risk, with IL-17A, TNF-related apoptosis-inducing ligand, and C–C motif chemokine 4 emerging as key inflammatory proteins. Notably, IL-17A was positively correlated with EMS progression, while TNF-related apoptosis-inducing ligand and C–C motif chemokine 4 exhibited protective effects. Mediation analysis further uncovered pathways where lipid metabolites modulate immune responses and inflammatory proteins, influencing EMS development. These findings suggest that lipid metabolism, immune traits, and inflammatory proteins may contribute to EMS pathogenesis, offering initial insights into potential mechanisms. Further experimental validation is needed to corroborate these results.

## 1. Introduction

Endometriosis (EMS) is a common gynecological disorder characterized by the ectopic growth of endometrial-like tissue outside the uterine cavity, accompanied by chronic inflammation. It affects approximately 10% of women of reproductive age worldwide. The major clinical manifestations include dysmenorrhea, chronic pelvic pain, infertility, and reduced quality of life, and in severe cases, the disease can impair social functioning and impose substantial psychological burden. Despite advances in elucidating the etiology and pathogenesis in recent years, EMS remains a complex disorder.^[1,2]^ Diagnosis still relies on invasive laparoscopic examination, while treatment mainly includes hormonal therapy and surgical resection, both of which are limited by suboptimal long-term efficacy, high recurrence rates, and adverse effects. These limitations underscore the urgent need for breakthroughs in mechanistic understanding, diagnostic modalities, and therapeutic strategies.^[3,4]^

Current research on EMS is transitioning from the exploration of single pathological mechanisms toward integrative multi-omics approaches and systems biology modeling. Accumulating evidence indicates that immune dysregulation, chronic inflammation, hormone dependence, metabolic abnormalities, and genetic susceptibility jointly contribute to the disease’s development and progression. Among these, aberrant immune activation is considered a crucial factor enabling ectopic endometrial cells to evade immune clearance, establish implantation, and promote lesion expansion.^[4]^ Frequent findings include macrophage dysfunction, imbalanced T cell subsets, and reduced natural killer (NK) cell activity, alongside elevated levels of inflammatory cytokines such as IL-6, TNF-α, and interleukin-17A (IL-17A), which further exacerbate the inflammatory microenvironment.^[5–8]^ However, most of these findings originate from observational studies, limiting the ability to infer causal relationships due to potential confounding and reverse causality.

In parallel, metabolic dysregulation (particularly involving lipid and glucose metabolism) has also been implicated in the persistence of EMS. Lipidomic and metabolomic studies have revealed widespread abnormalities in sphingolipids, ceramides, fatty acids, and energy metabolism in EMS patients.^[9]^ Upregulation of upstream regulatory genes such as *PDK1*, *HIF1A*, and *SMS1* suggests that metabolic imbalance may contribute to lesion formation by affecting cell adhesion, immune responses, and microenvironmental stability.^[10,11]^ Genome-wide association studies (GWAS) have identified multiple single nucleotide polymorphism (SNPs) associated with EMS, mainly in European and East Asian populations, yet these explain only a small portion of the estimated heritability.^[2]^ Most variants are located in non-coding regions, and their functional relevance remains unclear. Current evidence suggests that rare variants and epigenetic alterations (such as aberrant DNA methylation in endometrial tissues) may play critical roles. However, the lack of representation from non-European populations and limited integration with transcriptomic data hinder further mechanistic interpretation and clinical application.^[12–14]^ Moreover, the functional links between these genetic signals and immune or metabolic pathways remain unclear, hampering the translation of association findings into actionable mechanistic targets.

Beyond the mechanistic fragmentation and lack of hierarchical understanding, EMS research also faces significant challenges in diagnosis and treatment. Early diagnostic tools are lacking, with clinical diagnosis still relying on invasive laparoscopy with histological confirmation. Invasive laparoscopy with histological confirmation remains the clinical gold standard for diagnosis, yet significant diagnostic delays are commonly reported.^[15]^ Imaging techniques lack sensitivity in detecting early or small lesions and lack standardized evaluation criteria.^[15]^ Although biomarkers such as IL-6 and IL-17A have been proposed, their specificity is insufficient for standalone diagnostic use. Current hormonal therapies are associated with notable side effects, including progesterone resistance, and surgical interventions often result in recurrence. Fertility preservation remains a major clinical challenge.^[16]^

Importantly, limitations in research methodology further constrain the depth of mechanistic insights into EMS. Most studies adopt cross-sectional observational designs, which are inadequate for disentangling cause-and-effect relationships. Existing animal models fail to fully recapitulate the human phenotype, impeding clinical translation. Furthermore, the capacity to integrate interdisciplinary data is limited; the interrelationships among multi-omics datasets (such as immunophenotyping and lipidomic profiles) remain poorly characterized. Additionally, most genetic studies have focused on populations of European ancestry, while the underrepresentation of Asian women in public datasets hinders generalizability and the advancement of precision medicine.^[17,18]^ In summary, EMS is a multifactorial disease shaped by complex interactions among genetic, immunological, and metabolic factors. Although research is shifting from single-mechanism studies to integrative, multidimensional frameworks, significant barriers remain, including unclear causal mechanisms, unidentified key pathways, and methodological stagnation. Against this backdrop, novel analytical frameworks are urgently needed to overcome the limitations of traditional observational research and clarify the directional relationships among phenotypic features.

Mendelian randomization (MR) is an instrumental variable-based approach that leverages naturally occurring genetic variation to infer causal relationships between exposures and outcomes. By mimicking the random allocation of clinical trials, MR can assess the long-term effects of modifiable exposures on disease risk without requiring interventions, and has been increasingly applied in studies of metabolism, immunity, inflammation, and complex diseases.^[7,19]^

In this study, we constructed a two-step MR framework using large-scale GWAS datasets to systematically evaluate the causal chain linking lipid metabolism, immune cell traits, and inflammatory proteins in the etiology of EMS. Specifically, we aimed to determine whether lipid metabolism influences EMS risk via immune system modulation, distinguishing direct from mediated effects. This work provides mechanistic insights and causal evidence that may inform the development of targeted interventions for EMS.

## 2. Research objectives

**Figure 1.**
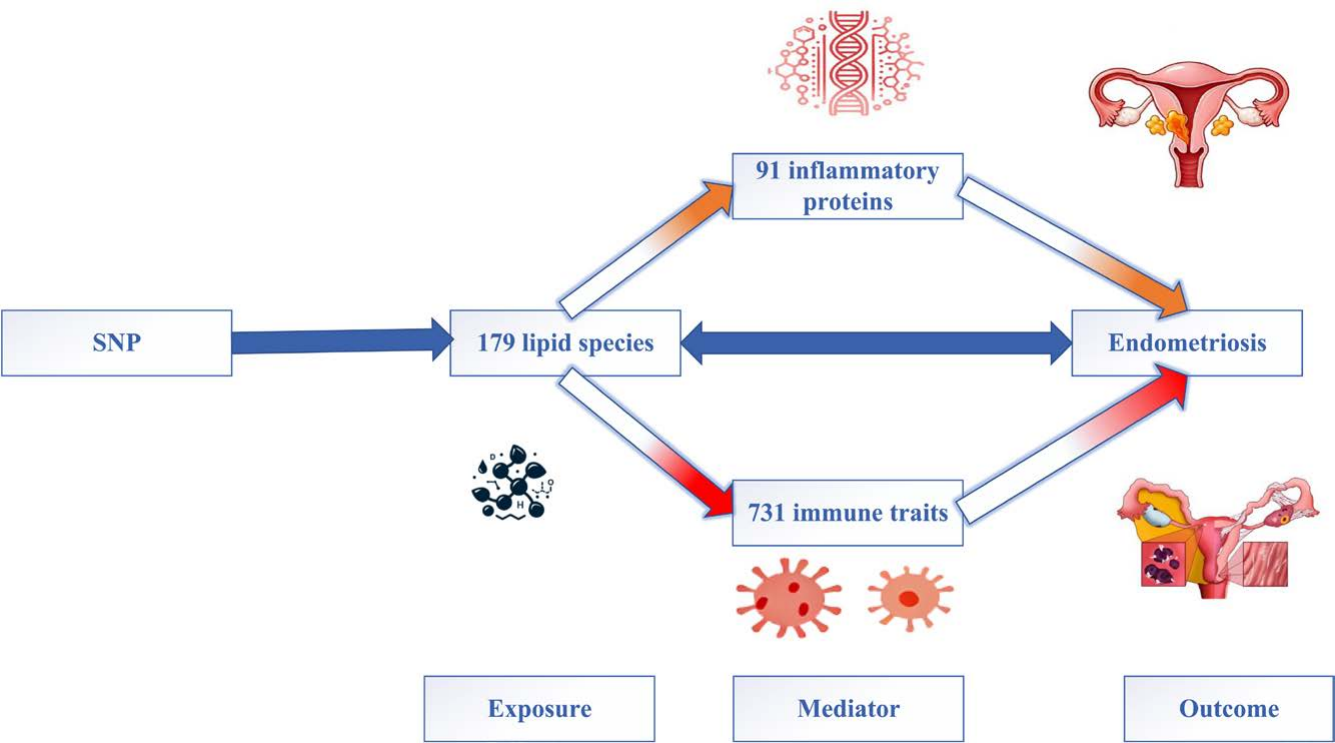
Integrative Mendelian randomization framework assessing the causal and mediating roles of lipid metabolism, immune traits, and inflammatory proteins in endometriosis. The analytic strategy comprises 4 components: (1) bidirectional Mendelian randomization to evaluate the causal relationship between 179 circulating lipid species and endometriosis; (2) univariable MR to estimate the independent effects of 731 immune cell traits and 91 inflammatory proteins on EMS risk; (3) forward MR assessing the regulatory influence of lipid species on immune and inflammatory mediators; and (4) two-step MR and mediation analysis to delineate whether immune traits and inflammatory proteins act as intermediaries in the lipid–EMS axis.

This study aims to investigate the causal relationships between lipid metabolism, immune cell traits, inflammatory proteins, and EMS through MR analysis. Specifically, it seeks to (1) assess the bidirectional causal effects of 179 lipid metabolic levels on EMS; (2) evaluate the independent causal effects of 731 immune cell traits and 91 inflammatory proteins on EMS to understand the immune system’s role in its pathogenesis; (3) explore the potential impact of lipid metabolism on immune cell traits and inflammatory proteins to elucidate lipid metabolism’s regulatory role on immune function and the inflammatory environment; and (4) conduct mediation analysis to determine whether immune cells and inflammatory proteins mediate the effect of lipid metabolism on EMS, distinguishing direct from indirect effects (Fig. 1).

### 2.1. Study population and data sources

This study utilizes publicly available data from GWAS. Lipid metabolism data were obtained from a 2023 GWAS conducted by Linda Ottensmann et al, which analyzed the plasma lipidome of 7174 Finnish individuals, covering 179 lipid metabolites and identifying 495 genetic variants associated with lipid metabolism (GWAS Catalog accession IDs: GCST90277249–GCST90277416).^[20]^ Immune cell trait data were derived from a study of 3757 individuals from Sardinia, evaluating the genetic characteristics of 731 immune cell traits and highlighting the significant connection between immune cell regulation and autoimmune diseases. The complete GWAS summary statistics are available in the GWAS Catalog (accession IDs: GCST0001391–GCST0002121).^[21]^ Data on inflammatory proteins were obtained from a 2023 study by Jing Hua Zhao and colleagues, which provided genetic and protein level information on 91 circulating inflammatory proteins, based on data from 14,824 individuals (GWAS Catalog accession IDs: GCST90274758–GCST90274848).^[22]^ EMS data were sourced from the Finnish FinnGen database (ID: finngen_R9_N14_ENDOMETRIOSIS), a large cohort study encompassing 15,088 female patients with an average age of 37.48 years. This dataset includes detailed case counts of EMS in various anatomical sites, such as 2929 cases in the pelvic peritoneum, 2747 in the ovaries, and 1807 in the uterus.^[23]^ All datasets used have received ethics committee approval, are publicly accessible, and do not involve personal privacy concerns (Table S1, Supplemental Digital Content, https://links.lww.com/MD/P371). This study included data from multiple GWAS (Our analysis used publicly available GWAS summary statistics. No new data were collected, and no new ethical approval was required.)

In MR analysis, the selection of instrumental variables (genetic instruments) is critical to the study’s success. Each instrumental variable for exposures (lipid metabolism levels and immune cell traits) must meet the following criteria: the instrumental variable should be strongly associated with the exposure (i.e., SNPs significantly associated in GWAS of lipid metabolism and immune cell traits), with a significance threshold of *P* < 5 × 10^−5^. The association between the instrumental variable and the exposure should not be influenced by potential confounders. Additionally, the instrumental variable should affect the outcome exclusively through the exposure, without any direct pathway to the outcome.^[24]^ To ensure independence among instrumental variables, we excluded SNPs in linkage disequilibrium with *r*^2^ > 0.001and a distance of < 10,000 kb. Furthermore, we used the PhenoScanner database to filter out SNPs associated with potential confounders, ensuring that the instrumental variables were only related to the exposure factors. Finally, F-statistics were calculated to retain SNPs with F > 10, enhancing the statistical power and robustness of the study.^[24,25]^

### 2.2. Statistical analysis

This study employed several methods for causal effect analysis, including the inverse variance weighted (IVW) method, Mendelian randomization Egger regression (MR-Egger), weighted median, and weighted mode approaches. The IVW method, used as the primary analytical tool, integrates the effects of all genetic instruments using weighted least squares to enhance statistical power and provide precise causal effect estimates. However, to ensure the robustness of the results, MR-Egger analysis was also conducted to assess the presence of horizontal pleiotropy among instrumental variables, identifying potential biases due to unadjusted pleiotropic effects.^[26,27]^ Additionally, the weighted median and weighted mode methods were applied as robustness checks; these methods can yield stable causal effect estimates even if some instruments are biased.^[28]^

This study estimated mediation effects using the difference method and the product of coefficients method.^[29,30]^ MR-Egger regression was applied to assess horizontal pleiotropy, with the intercept indicating potential influences of instrumental variables on the outcome through pathways other than the exposure. The weighted median and weighted mode methods were used for supplementary analysis, offering reliable estimates even if some instruments were invalid. Cochran Q statistic tested heterogeneity among instrumental variables. These sensitivity analyses ensured the robustness of the MR findings.^[31]^

## 3. Results

### 3.1. Bidirectional MR analysis of lipid metabolites and EMS

**Figure 2.**
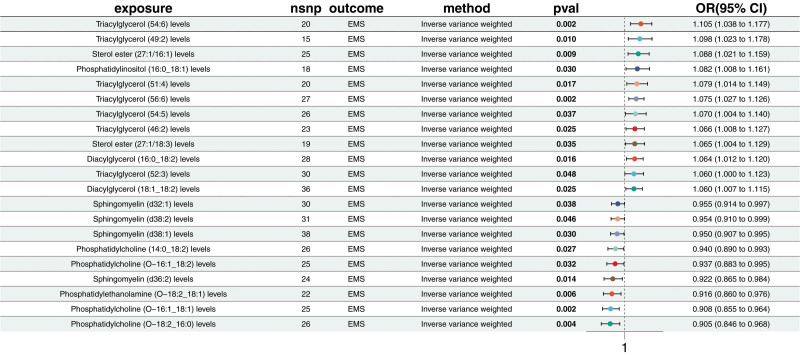
Forest plot of Mendelian randomization estimates for the causal effects of lipid metabolites on endometriosis. Causal estimates were derived using the inverse variance weighted method. Odds ratios and 95% confidence intervals are shown. All associations presented reached nominal statistical significance (*P* < .05).

Through MR analysis, this study identified significant associations between 21 lipid metabolic levels and EMS (Fig. 2). These included 5 types of phosphatidylcholine (PC), one type of phosphatidylethanolamine (PE), 4 types of sphingomyelin (SM), 2 types of diacylglycerol (DAG), 7 types of triacylglycerol (TG), 3 types of sterol ester (SE), and 1 type of phosphatidylinositol. Of these, 9 metabolites were negatively associated with EMS, suggesting potential protective effects. These include PC (O-18:2_18:2) levels (odds ratio, OR = 0.89; confidence interval [CI] = 0.809–0.979; *P* = .016), PC (O-18:2_16:0) levels (OR = 0.905; CI = 0.846–0.968; *P* = .004), PC (O-16:1_18:1) levels (OR = 0.908; CI = 0.855–0.964; *P* = .002), PE (O-18:2_18:1) levels (OR = 0.916; CI = 0.86–0.976; *P* = .006), SM (d36:2) levels (OR = 0.922; CI = 0.865–0.984; *P* = .014), and others. Conversely, 12 lipid metabolic levels were positively associated with EMS, indicating potential risk factors. These include DAG (18:1_18:2) levels (OR = 1.06; CI = 1.007–1.115; *P* = .025), TG (52:3) levels (OR = 1.06; CI = 1–1.123; *P* = .048), DAG (16:0_18:2) levels (OR = 1.064; CI = 1.012–1.12; *P* = .016), SE (27:1/18:3) levels (OR = 1.065; CI = 1.004–1.129; *P* = .035), TG (46:2) levels (OR = 1.066; CI = 1.008–1.127; *P* = .025), and others (Table S2, Supplemental Digital Content, https://links.lww.com/MD/P371). When the disease was used as the exposure and 179 lipid metabolites were used as the outcomes for reverse MR to evaluate the causal relationship between lipid metabolites and EMS, it was found that 178 lipid metabolites showed no reverse causal relationship with EMS. However, TG (46:2) levels exhibited a bidirectional causal relationship (Fig. 2 and Table S4, Supplemental Digital Content, https://links.lww.com/MD/P371). Subsequently, TG (46:2) levels were excluded from the mediation analysis. This analysis did not detect pleiotropy or heterogeneity (Table S3 and Table S5, Supplemental Digital Content, https://links.lww.com/MD/P371).

#### 3.1.1. MR analysis of immune cell traits and EMS

**Figure 3.**
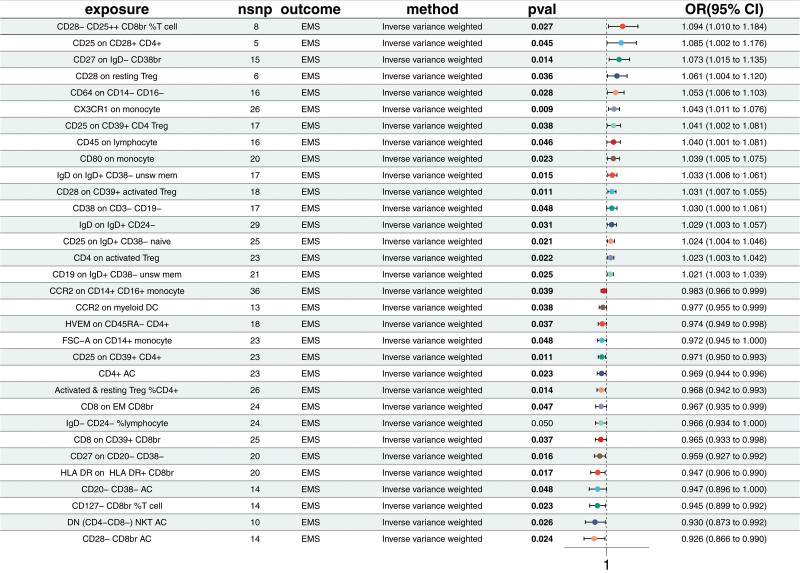
Forest plot of Mendelian randomization estimates for the causal effects of immune cell traits on endometriosis. Causal estimates were obtained using the inverse variance weighted method. Odds ratios and 95% confidence intervals are shown. A total of 32 immune traits showed significant associations with endometriosis (*P* < .05), including both protective and risk-enhancing effects.

The MR analysis revealed significant associations between 32 immune cell traits and EMS. Sixteen traits were negatively associated with EMS, suggesting potential protective roles. These include CD28- CD8br AC (OR = 0.926; CI = 0.866–0.99; *P* = .024), DN (CD4- CD8-) NKT AC (OR = 0.93; CI = 0.873–0.992; *P* = .026), CD127- CD8br %T cell (OR = 0.945; CI = 0.899–0.992; *P* = .023), and others. In contrast, 16 traits were positively associated with EMS, indicating potential contributing factors in EMS pathogenesis. These include CD28 on CD39 + activated regulatory T cells (Treg) (OR = 1.031; CI = 1.007–1.055; *P* = .011), CD19 on IgD + CD38- unsw mem (OR = 1.021; CI = 1.003–1.039; *P* = .025), and others (Fig. 3 and Table S6, Supplemental Digital Content, https://links.lww.com/MD/P371). This analysis did not detect pleiotropy or heterogeneity (Table S7, Supplemental Digital Content, https://links.lww.com/MD/P371).

#### 3.1.2. MR analysis of 91 inflammatory proteins and EMS

**Figure 4.**
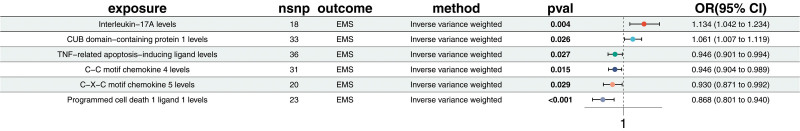
Forest plot of Mendelian randomization estimates for the causal effects of inflammatory proteins on endometriosis. Causal estimates were derived using the inverse variance weighted method. Odds ratios and 95% confidence intervals are shown. Six inflammatory proteins demonstrated significant associations with endometriosis (*P* < .05), including both risk-enhancing and protective effects.

Using the IVW method, 6 inflammatory proteins were found to be associated with EMS. Two inflammatory proteins were positively associated with EMS, indicating that higher levels may increase EMS risk. These include IL-17A (OR = 1.134; CI = 1.042–1.234; *P* = .004), and CUB domain-containing protein 1 (OR = 1.061; CI = 1.007–1.119; *P* = .026). Four inflammatory proteins were negatively associated with EMS, suggesting a potential protective role. These include TNF-related apoptosis-inducing ligand (TRAIL) (OR = 1.061; CI = 1.007–1.119; *P* = .026), C–C motif chemokine 4 (OR = 0.946; CI = 0.904–0.989; *P* = .015), and others (Fig. 4 and Table S8, Supplemental Digital Content, https://links.lww.com/MD/P371). This analysis did not detect pleiotropy or heterogeneity (Table S9, Supplemental Digital Content, https://links.lww.com/MD/P371).

### 3.2. Mediator screening

**Figure 5.**
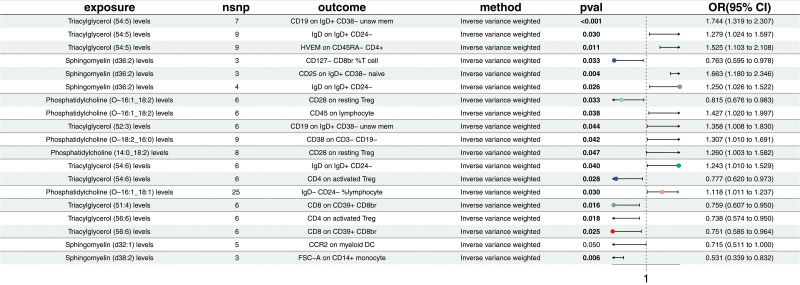
Effect proportions mediated by immune traits in the causal pathway from lipid metabolites to endometriosis. The mediation analysis was conducted using a two-step Mendelian randomization framework. Bars represent the proportion of the total effect of each lipid metabolite on endometriosis that is mediated through specific immune cell traits. Only statistically significant mediation pathways (*P* < .05) are shown.

**Figure 6.**
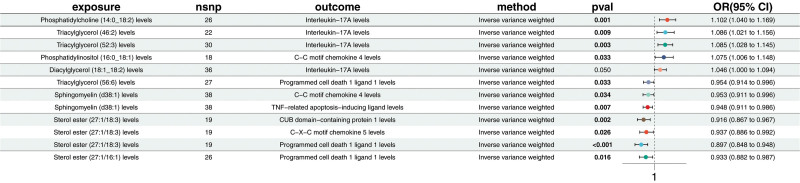
Effect proportions mediated by inflammatory proteins in the causal pathway from lipid metabolites to endometriosis. Mediation analysis was performed using a two-step Mendelian randomization approach. Bars indicate the proportion of the total effect of lipid metabolites on endometriosis that is mediated through specific inflammatory proteins. Only statistically significant mediation effects (*P* < .05) are presented.

After identifying the impact of lipid metabolites and immune cell traits on EMS, the study further analyzed 20 lipid metabolites as exposures and 32 immune cell traits as outcomes using MR. Nineteen causal relationships were identified (Fig. 5 and Table S10, Supplemental Digital Content, https://links.lww.com/MD/P371). Additionally, 20 lipid metabolites were used as exposures and 6 inflammatory proteins as outcomes in the MR analysis, resulting in the identification of 12 causal relationships (Fig. 6 and Table S11, Supplemental Digital Content, https://links.lww.com/MD/P371). The analysis did not indicate any pleiotropy or heterogeneity.

#### 3.2.1. Mediation analysis

**Figure 7.**
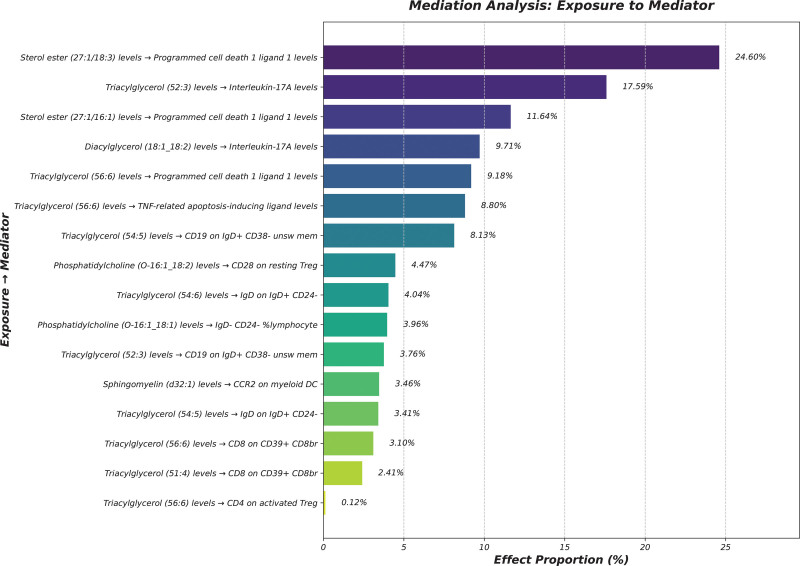
Combined effect proportions mediated by immune traits and inflammatory proteins in the pathway from lipid metabolites to endometriosis. This bar plot summarizes significant mediation pathways identified through two-step Mendelian randomization analysis. Each bar represents the proportion of the total effect of a lipid metabolite on endometriosis that is mediated through a specific immune or inflammatory mediator. The top mediating factors include programmed cell death 1 ligand 1, interleukin-17A, CD19 on IgD⁺ CD38⁻ unswitched memory B cells, and others.

After identifying the key mediators influencing EMS and assessing the subsequent impact of exposures on these mediators, this study quantified the mediation effects. The indirect effects were calculated by subtracting the direct effects from the total effects, with the direct effects being evaluated based on the direct influence of lipid metabolites (Supplementary Table, Supplemental Digital Content, https://links.lww.com/MD/P371). Specifically, SE (27:1/18:3) levels were observed to influence immune responses through programmed cell death 1 ligand 1 and TRAIL, with effect sizes of 24.60% and 17.77%, respectively. TG (52:3) levels showcased notable mediation effects on immune regulation via IL-17A, with an effect size of 17.59% (*P* = .003) (Fig. 7, Table S12 and S13, Supplemental Digital Content, https://links.lww.com/MD/P371). These results highlight the complex interactions between lipid metabolites, immune traits, and inflammatory proteins in influencing EMS, providing deeper insights into the pathways involved.

## 4. Discussion

To date, this study represents the first to use multi-omics data combined with MR to investigate the causal relationships between 179 lipid metabolic levels, 731 immune phenotypes, 91 inflammatory proteins, and EMS. It also explores possible mediation pathways and effects, potentially offering novel therapeutic targets for EMS diagnosis and treatment.

In recent years, lipid metabolites have attracted increasing attention in the context of EMS due to their potential involvement in disease pathogenesis. Previous studies have demonstrated significant alterations in multiple lipid species (including PC, PE, phosphatidylinositol, DAG, TG, and SE) in patients with EMS.^[32–34]^ Mass spectrometry analyses in EMS animal models have further identified abnormal accumulation of these lipids in ectopic lesions, suggesting their possible role in disease progression.^[35]^ In the present study, we conducted a comprehensive MR analysis to assess causal relationships between 179 lipid metabolites and EMS. Our findings revealed positive associations between several glycerolipid-related metabolites (including DAG, TG, and SE) and increased EMS risk, while selected subclasses of PC, PE, and SM were negatively associated, indicating that lipid metabolism may contribute to both disease susceptibility and defense through distinct pathways.

The impact of lipid metabolic dysregulation in EMS extends beyond abnormal metabolite accumulation. Lipids are functionally involved in inflammation modulation, immune regulation, fatty acid oxidation, hormone signaling, and autophagy.^[36]^ In particular, disturbances in sphingolipid metabolism have been implicated in autophagy-related dysfunction, which may accelerate lesion development.^[35]^ Sphingolipid-derived metabolites influence membrane fluidity and intracellular signaling, thereby affecting the local microenvironment of ectopic lesions. Studies have shown altered levels of fatty acids such as palmitic acid and docosahexaenoic acid in granulosa cells of EMS patients, accompanied by decreased sphingosine levels in mural granulosa cells. These changes may impair follicular function and oocyte quality, potentially contributing to infertility.^[37,38]^

At the molecular level, upregulation of key genes involved in sphingolipid synthesis (such as CERS1, SPTL1, and SMPD1) has been associated with enhanced autophagy and cellular stress responses. Conversely, downregulation of enzymes such as SPHK1, ASAH1, and SGPP1 in mural granulosa cells, along with elevated expression of CERS1 and UGCG, suggests that this pathway may be linked to localized chronic inflammation and oxidative stress.^[37,39]^

The relationship between lipid metabolism and endocrine signaling is also of increasing interest. A positive correlation has been reported between the triglyceride-glucose (TyG) index and EMS risk, supporting the hypothesis that disrupted insulin signaling may affect DAG and TG metabolism and impair lipid homeostasis.^[40]^ Insulin resistance has been shown to enhance aromatase activity, promoting the conversion of androgens to estrogens and triggering inflammatory cascades via estrogen receptor alpha activation. This pathway contributes to immune cell recruitment and sustained inflammatory responses.^[41,42]^ Animal and clinical studies further confirm that estrogen receptor alpha deficiency attenuates lesion formation, whereas elevated estrogen levels are associated with increased activation of mast cells, macrophages, and neutrophils.^[43–45]^ Our MR analysis reinforces this mechanistic axis by demonstrating a robust causal relationship between TG levels and EMS risk.

In addition, the lipid species positively associated with EMS in this study were predominantly DAG, TG, and SM, consistent with their established roles in inflammatory activation. DAG and TG promote the expression of pro-inflammatory cytokines such as TNF-α and stimulate lipolysis, leading to increased levels of free fatty acids that further amplify inflammation.^[37]^ Sphingolipids and lysophospholipids also contribute to mitochondrial dysfunction, NLRP3 inflammasome activation, and autophagy induction, reinforcing cellular stress and chronic inflammation through positive feedback mechanisms.^[46,47]^

Multiple studies have highlighted the close association between EMS pathogenesis and immune system dysregulation, particularly involving immune cells like Treg, monocytes, macrophages, and B cells.^[48,49]^ Our findings indicate a significant association between various Treg subpopulations and EMS. In EMS patients, the proportion of Treg cells in peripheral blood and endometrial tissue, particularly activated CD4 + Treg cells, is markedly reduced, alongside decreased expression of key genes such as CD25, FOXP3, and CTLA-4, with FOXP3 deficiency closely linked to peritoneal lesion formation.^[45,50,51]^ Treg dysfunction weakens the secretion of inhibitory factors like IL-10 and TGF-β, while increasing the expression of pro-inflammatory factors such as TNF-α and IL-6, thereby promoting lesion expansion and immune evasion.^[52]^ Clinical studies show an inverse correlation between Treg cell counts and EMS severity, with Treg markers (e.g., CD25 and CD39) significantly decreasing as the disease progresses.^[53]^

Additionally, macrophage overactivation leads to the release of pro-inflammatory factors like IL-6 and VEGF, driving local inflammation and angiogenesis, which are integral to EMS’s chronic inflammation and lesion formation.^[54,55]^ B cell aggregation, especially in lymphoid clusters within ectopic endometrial tissue, affects EMS pathology through antigen clearance and antibody production.^[56]^ NK cell dysfunction, marked by reduced activation receptors and increased inhibitory receptors, is also associated with compromised immune surveillance, reducing the clearance of ectopic endometrial cells.^[57]^ Overall, immune dysregulation in EMS involves multiple cell types and pathways, with this complex regulatory imbalance accelerating lesion progression and chronicity. This study refines our understanding of specific roles for different Treg and macrophage subpopulations and other immune cells, offering a theoretical foundation for targeted immunomodulatory therapies in EMS. This aligns with our research.

Inflammatory responses play a role throughout EMS’s development and regression. By modifying the immune environment, inflammation can activate immune evasion mechanisms, driving EMS progression. In this study, pro-inflammatory factors such as IL-17A were positively associated with EMS, playing critical roles in the inflammatory process. The number of Th17 cells and the level of IL-17A are significantly increased in the peritoneal fluid of patients with EMS. IL-17A promotes the survival of endometrial cells by upregulating the expression of anti-apoptotic genes such as Bcl-2 and MCL1, and enhances their survival through the activation of the ERK1/2 signaling pathway. Additionally, IL-17A inhibits NK cell-mediated cytotoxicity and induces the expression of HLA-G on endometrial cells, thereby helping these cells evade immune surveillance.^[58,59]^ Conversely, some inflammatory proteins, such as TRAIL, were negatively associated with EMS, correlating with reduced disease risk. As a pro-apoptotic factor, elevated TRAIL levels may reduce EMS lesion expansion by promoting cell apoptosis.^[60]^ Studies indicate that TRAIL levels are significantly lower in peritoneal fluid of EMS patients, particularly in advanced stages, where a decrease in the TRAIL/osteoprotegerin ratio suggests that elevated osteoprotegerin may counteract TRAIL’s pro-apoptotic effects, supporting lesion persistence.^[61,62]^

## 5. Strengths and Limitations

The reliance on publicly available GWAS summary data precluded the inclusion of individual-level covariates and interaction analyses, and the population specificity to European cohorts limits generalizability to other ethnic groups. Although sensitivity analyses, including MR-Egger and MR-PRESSO, were applied to address pleiotropy, residual pleiotropic effects cannot be entirely excluded. The assumptions of MR (relevance, independence, and exclusion restriction) are inherently unverifiable and could potentially bias the causal estimates if violated. Mediation analysis was constrained by the resolution of genetic instruments and excluded lipid metabolites with bidirectional causal relationships, such as TG (46:2), which may have restricted the scope of pathway exploration. Finally, the findings are derived from statistical associations and require experimental validation to substantiate the inferred causal relationships and underlying mechanisms. These limitations highlight the need for further investigation to refine and validate the observed associations.

## 6. Conclusion

This study applied MR to explore the roles of lipid metabolism, immune dysregulation, and inflammation in EMS. Significant associations were found between lipid metabolites, including DAG, TG, and SM, and EMS risk, suggesting lipid metabolism influences disease onset. Immune cell traits and inflammatory proteins such as IL-17A and TRAIL were also linked to EMS, indicating their involvement in the disease’s pathophysiology. Mediation analysis revealed pathways where lipid metabolites affect immune responses and inflammation, providing potential therapeutic targets. While these findings are based on statistical associations, experimental validation is needed to confirm causality. Future research should focus on validating these relationships and exploring their clinical relevance.

## Acknowledgments

All authors thank the patients and sequencers who provided samples and the publicly available databases.

## Author contributions

**Conceptualization:** Pei Guo.

**Data curation:** Pei Guo, Lin Xu.

**Formal analysis:** Pei Guo.

**Investigation:** Weihong Li.

**Project administration:** Weihong Li.

**Resources:** Junhong Gan.

**Software:** Junhong Gan.

**Supervision:** Weihong Li.

**Visualization:** Junhong Gan.

**Writing – original draft:** Pei Guo, Junhong Gan, Lin Xu.

**Writing – review & editing:** Weihong Li.

## Supplementary Material

SUPPLEMENTARY MATERIAL
